# 1-{2-Hy­droxy-6-[3-(pyrrol-1-yl)prop­oxy]phen­yl}ethanone

**DOI:** 10.1107/S1600536812010641

**Published:** 2012-03-17

**Authors:** Ali Ourari, Djouhra Aggoun, Sofiane Bouacida

**Affiliations:** a Laboratoire d’Electrochimie, d’Ingénierie Moléculaire et de Catalyse Redox (LEIMCR), Faculté des Sciences de l’Ingénieur, Université Farhat Abbas, Sétif 19000, Algeria; b Unité de Recherche de Chimie de l’Environnement et Moléculaire Structurale, CHEMS, Université Mentouri-Constantine, 25000 Algeria

## Abstract

In the title compound, C_15_H_17_NO_3_, the mean planes of the pyrrole and benzene rings form a dihedral angle of 81.92 (7)°. The mol­ecule contains an intra­molecular O—H⋯O hydrogen bond. In the crystal, weak C—H⋯π inter­actions link the mol­ecules into chains along [010].

## Related literature

For the synthesis and applications of similar compounds and their derivatives, see: Wu & Lu (2003 ▶); Saraswat *et al.* (2006 ▶); Smith *et al.* (2003 ▶); Dong *et al.* (2010 ▶); Deronzier & Moutet (1996 ▶); MacDearmid (2001 ▶); Srinivasan *et al.* (1986 ▶); Coche-Guerente *et al.* (1995 ▶); Ourari *et al.* (2008 ▶); Khedkar & Radhakrishnan (1997 ▶); Huo *et al.* (1999 ▶).
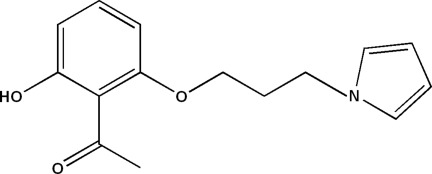


## Experimental

### 

#### Crystal data

C_15_H_17_NO_3_
*M* *_r_* = 259.3
Triclinic, 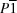

*a* = 7.741 (2) Å
*b* = 9.230 (1) Å
*c* = 10.464 (1) Å
α = 71.63 (2)°
β = 75.222 (1)°
γ = 82.081 (1)°
*V* = 684.7 (2) Å^3^
*Z* = 2
Mo *K*α radiation
μ = 0.09 mm^−1^
*T* = 295 K
0.15 × 0.08 × 0.04 mm

#### Data collection

Nonius KappaCCD diffractometer
4238 measured reflections
2586 independent reflections
1995 reflections with *I* > 2σ(*I*)
*R*_int_ = 0.020

#### Refinement

*R*[*F* ^2^ > 2σ(*F* ^2^)] = 0.046
*wR*(*F* ^2^) = 0.128
*S* = 1.05
2586 reflections
176 parameters
H atoms treated by a mixture of independent and constrained refinement
Δρ_max_ = 0.18 e Å^−3^
Δρ_min_ = −0.17 e Å^−3^

### 

Data collection: *COLLECT* (Nonius, 1998 ▶); cell refinement: *SCALEPACK* (Otwinowski & Minor, 1997 ▶); data reduction: *DENZO* (Otwinowski & Minor, 1997 ▶) and *SCALEPACK*; program(s) used to solve structure: *SIR2002* (Burla *et al.*, 2005 ▶); program(s) used to refine structure: *SHELXL97* (Sheldrick, 2008 ▶); molecular graphics: *ORTEP-3 for Windows* (Farrugia, 1997 ▶) and *DIAMOND* (Brandenburg & Berndt, 2001 ▶); software used to prepare material for publication: *WinGX* (Farrugia, 1999 ▶).

## Supplementary Material

Crystal structure: contains datablock(s) global, I. DOI: 10.1107/S1600536812010641/lh5428sup1.cif

Structure factors: contains datablock(s) I. DOI: 10.1107/S1600536812010641/lh5428Isup2.hkl

Supplementary material file. DOI: 10.1107/S1600536812010641/lh5428Isup3.cml

Additional supplementary materials:  crystallographic information; 3D view; checkCIF report

## Figures and Tables

**Table 1:** Hydrogen-bond geometry (Å, °) *Cg* is is the centroid of the N1/C12–C15 ring.

*D*—H⋯*A*	*D*—H	H⋯*A*	*D*⋯*A*	*D*—H⋯*A*
O3—H3⋯O2	0.98 (2)	1.578 (19)	2.498 (2)	153.4 (18)
C5—H5⋯*Cg*^i^	0.93	2.90	3.641 (2)	138
C11—H11*B*⋯*Cg*^ii^	0.97	2.74	3.3973 (19)	125

Symmetry codes: (i) 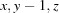; (ii) 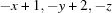.

**Fig. 1.**
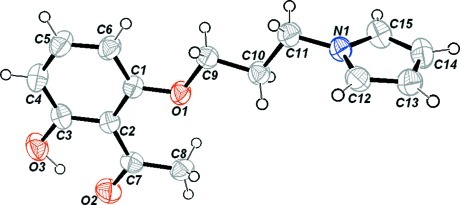
The molecular structure of (I) with displacement ellipsoids drawn at the 50% probability level. H atoms are represented as small spheres of arbitrary radii.

**Fig. 2.**
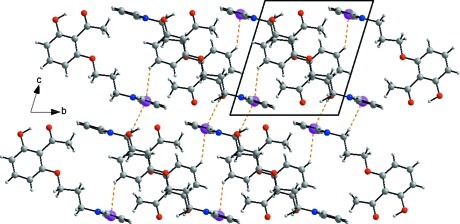
The packing showing weak C—H···π interactions involving the centroid (in pink) of the pyrrole ring as dashed lines.

## supplementary crystallographic information

### Comment

The synthesis of new derivatives containing both a pyrrole ring and salicyaldehyde moiety is of a great interest since they are currently used as precursors for chelating agents especially those of Schiff bases (Wu *et al.*, 2003; Saraswat *et al.*, 2006) and oximes (Smith *et al.*, 2003; Dong *et al.*, 2010). These compounds may also be involved in the elaboration of modified electrodes by anodic (Deronzier & Moutet, 1996) or by chemical oxidation (MacDearmid *et al.*, 2001). These types of materials can be applied in catalysis, electrocatalysis and sensors (Srinivasan *et al.*, 1986; Coche-Guerente *et al.*, 1995; Ourari *et al.*, 2008). The synthesis of new salicylaldehyde derivatives containing electropolymerizable units can be considered as the main source of a functionalized conducting π-conjugated polymers such as as those of polypyrrole and polyaniline (Khedkar *et al.*, 1997; Huo *et al.*, 1999).

We report herein the crystal structure of the title compound. The molecular structure is shown in Fig. 1. The mean planes of the pyrrole and benzene rings form a dihedral angle of 81.92 (7)°. There is an intramolecular O—H···O hydrogen bond present. In the crystal, there are weak C—H···π interactions (Table 1) which form chains of dimers along [010] (Fig. 2).

### Experimental

A solution of 152 mg (1 mmol) of 2,6-dihydroxyacetophenone was added to a solution containing 187 mg (1 mmol) of 1-bromopropyl-3-N-pyrrol and 181 mg (1.7 mmol) of potassium carbonate under argon atmosphere. The mixture was refluxed for 45 h and was allowed to stand at room temperature. After extraction by dichloromethane and purification by chromatography on silica gel using dichloromethane as eluent. Thus, 153 mg of pure compound (I) was recovered, corresponding to the yield of 59%. The suitable single crystals were then obtained from dichloromethane solution by slow evaporation.

### Refinement

H atoms were located in difference Fourier maps but introduced in calculated positions and treated as riding on their parent atoms (C) with C—H = 0.96 Å (methyl), 0.97 Å (methylene) or 0.93 Å (aromatic) with *U*_iso_(H) = 1.2*U*_eq_(C_aromatic_ and C_methylene_) or *U*_iso_(H) = 1.5*U*_eq_(C_methyl_). Atom H3 was located in a difference Fourier map and refined with *U*_iso_(H) = 1.2*U*_eq_ (O)

### Crystal data

**Table:**

C_15_H_17_NO_3_	*Z* = 2
*M**_r_* = 259.3	*F*(000) = 276
Triclinic, *P*1	*D*_x_ = 1.258 Mg m^−^^3^
*a* = 7.741 (2) Å	Mo *K*α radiation, λ = 0.71073 Å
*b* = 9.230 (1) Å	Cell parameters from 2211 reflections
*c* = 10.464 (1) Å	θ = 1.0–26.4°
α = 71.63 (2)°	µ = 0.09 mm^−^^1^
β = 75.222 (1)°	*T* = 295 K
γ = 82.081 (1)°	Plate, white
*V* = 684.7 (2) Å^3^	0.15 × 0.08 × 0.04 mm

### Data collection

**Table:**

Nonius KappaCCD diffractometer	1995 reflections with *I* > 2σ(*I*)
Radiation source: Enraf Nonius FR590	*R*_int_ = 0.020
Graphite monochromator	θ_max_ = 26.4°, θ_min_ = 3.1°
Detector resolution: 9 pixels mm^-1^	*h* = −8→8
CCD rotation images, thick slices scans	*k* = −11→11
4238 measured reflections	*l* = −11→13
2586 independent reflections	

### Refinement

**Table:**

Refinement on *F*^2^	Primary atom site location: structure-invariant direct methods
Least-squares matrix: full	Secondary atom site location: difference Fourier map
*R*[*F*^2^ > 2σ(*F*^2^)] = 0.046	Hydrogen site location: inferred from neighbouring sites
*wR*(*F*^2^) = 0.128	H atoms treated by a mixture of independent and constrained refinement
*S* = 1.05	*w* = 1/[σ^2^(*F*_o_^2^) + (0.0614*P*)^2^ + 0.0857*P*] where *P* = (*F*_o_^2^ + 2*F*_c_^2^)/3
2586 reflections	(Δ/σ)_max_ < 0.001
176 parameters	Δρ_max_ = 0.18 e Å^−^^3^
0 restraints	Δρ_min_ = −0.17 e Å^−^^3^

### Special details

**Table:**

Geometry. All e.s.d.'s (except the e.s.d. in the dihedral angle between two l.s. planes) are estimated using the full covariance matrix. The cell e.s.d.'s are taken into account individually in the estimation of e.s.d.'s in distances, angles and torsion angles; correlations between e.s.d.'s in cell parameters are only used when they are defined by crystal symmetry. An approximate (isotropic) treatment of cell e.s.d.'s is used for estimating e.s.d.'s involving l.s. planes.
Refinement. Refinement of *F*^2^ against ALL reflections. The weighted *R*-factor *wR* and goodness of fit *S* are based on *F*^2^, conventional *R*-factors *R* are based on *F*, with *F* set to zero for negative *F*^2^. The threshold expression of *F*^2^ > σ(*F*^2^) is used only for calculating *R*-factors(gt) *etc*. and is not relevant to the choice of reflections for refinement. *R*-factors based on *F*^2^ are statistically about twice as large as those based on *F*, and *R*- factors based on ALL data will be even larger.

### Fractional atomic coordinates and isotropic or equivalent isotropic displacement parameters (Å^2^)

**Table:**

	*x*	*y*	*z*	*U*_iso_*/*U*_eq_	
C1	0.22766 (19)	0.41969 (15)	0.52869 (14)	0.0461 (3)	
C2	0.13789 (18)	0.35148 (15)	0.66752 (14)	0.0462 (3)	
C3	0.1027 (2)	0.19569 (17)	0.70372 (16)	0.0543 (4)	
C4	0.1559 (2)	0.11311 (18)	0.60838 (19)	0.0641 (4)	
H4	0.1327	0.0104	0.6342	0.077*	
C5	0.2426 (2)	0.18421 (18)	0.47626 (18)	0.0653 (5)	
H5	0.278	0.1286	0.4126	0.078*	
C6	0.2790 (2)	0.33656 (17)	0.43500 (16)	0.0571 (4)	
H6	0.3378	0.3828	0.3445	0.069*	
C7	0.0815 (2)	0.43219 (18)	0.77474 (15)	0.0523 (4)	
C8	0.1265 (2)	0.59134 (19)	0.75300 (18)	0.0631 (4)	
H8A	0.0639	0.6615	0.6879	0.095*	
H8B	0.2531	0.5999	0.7177	0.095*	
H8C	0.0914	0.6152	0.8393	0.095*	
C9	0.3468 (2)	0.64362 (16)	0.35422 (14)	0.0503 (4)	
H9A	0.2802	0.633	0.2911	0.06*	
H9B	0.4663	0.5963	0.3328	0.06*	
C10	0.3574 (2)	0.81022 (16)	0.33794 (15)	0.0503 (4)	
H10A	0.4256	0.8209	0.4001	0.06*	
H10B	0.2381	0.8572	0.3608	0.06*	
C11	0.4473 (2)	0.88841 (17)	0.19002 (16)	0.0605 (4)	
H11A	0.5719	0.851	0.1736	0.073*	
H11B	0.3915	0.8598	0.1291	0.073*	
C12	0.2945 (2)	1.14975 (17)	0.12267 (16)	0.0559 (4)	
H12	0.1838	1.1193	0.1252	0.067*	
C13	0.3403 (2)	1.29658 (18)	0.08711 (17)	0.0612 (4)	
H13	0.2669	1.3843	0.0614	0.073*	
C14	0.5179 (3)	1.29123 (19)	0.09628 (18)	0.0657 (5)	
H14	0.5847	1.3749	0.0775	0.079*	
C15	0.5757 (2)	1.14127 (19)	0.13775 (17)	0.0621 (4)	
H15	0.6892	1.1045	0.1524	0.074*	
N1	0.43895 (17)	1.05482 (13)	0.15398 (12)	0.0517 (3)	
O1	0.25933 (15)	0.57023 (11)	0.49358 (10)	0.0550 (3)	
O2	−0.00716 (18)	0.36498 (15)	0.89029 (12)	0.0776 (4)	
O3	0.01756 (18)	0.11918 (14)	0.83236 (13)	0.0736 (4)	
H3	−0.005 (3)	0.197 (2)	0.882 (2)	0.088*	

### Atomic displacement parameters (Å^2^)

**Table:**

	*U*^11^	*U*^22^	*U*^33^	*U*^12^	*U*^13^	*U*^23^
C1	0.0511 (8)	0.0426 (7)	0.0465 (8)	−0.0031 (6)	−0.0141 (6)	−0.0132 (6)
C2	0.0462 (8)	0.0477 (8)	0.0460 (8)	−0.0038 (6)	−0.0141 (6)	−0.0121 (6)
C3	0.0558 (9)	0.0518 (8)	0.0538 (9)	−0.0111 (6)	−0.0175 (6)	−0.0059 (7)
C4	0.0801 (12)	0.0467 (8)	0.0701 (11)	−0.0104 (7)	−0.0237 (9)	−0.0155 (8)
C5	0.0868 (13)	0.0530 (9)	0.0645 (10)	−0.0031 (8)	−0.0200 (9)	−0.0269 (8)
C6	0.0730 (11)	0.0512 (8)	0.0489 (9)	−0.0053 (7)	−0.0107 (7)	−0.0188 (7)
C7	0.0480 (8)	0.0637 (9)	0.0467 (8)	−0.0041 (6)	−0.0105 (6)	−0.0180 (7)
C8	0.0653 (10)	0.0705 (11)	0.0600 (10)	−0.0061 (8)	−0.0056 (7)	−0.0340 (8)
C9	0.0562 (9)	0.0495 (8)	0.0430 (8)	−0.0050 (6)	−0.0084 (6)	−0.0118 (6)
C10	0.0575 (9)	0.0465 (8)	0.0455 (8)	−0.0052 (6)	−0.0110 (6)	−0.0111 (6)
C11	0.0778 (11)	0.0459 (8)	0.0492 (9)	−0.0042 (7)	−0.0038 (7)	−0.0103 (7)
C12	0.0514 (9)	0.0570 (9)	0.0536 (9)	−0.0068 (7)	−0.0066 (6)	−0.0108 (7)
C13	0.0689 (11)	0.0508 (9)	0.0560 (9)	−0.0013 (7)	−0.0079 (7)	−0.0105 (7)
C14	0.0820 (12)	0.0549 (9)	0.0598 (10)	−0.0214 (8)	−0.0160 (8)	−0.0093 (8)
C15	0.0612 (10)	0.0631 (10)	0.0592 (10)	−0.0127 (7)	−0.0178 (7)	−0.0068 (8)
N1	0.0588 (8)	0.0448 (7)	0.0453 (7)	−0.0061 (5)	−0.0065 (5)	−0.0076 (5)
O1	0.0754 (7)	0.0439 (6)	0.0426 (6)	−0.0116 (5)	−0.0041 (5)	−0.0123 (4)
O2	0.0910 (9)	0.0849 (9)	0.0502 (7)	−0.0214 (7)	0.0058 (6)	−0.0209 (6)
O3	0.0885 (9)	0.0633 (8)	0.0604 (8)	−0.0263 (6)	−0.0079 (6)	−0.0040 (6)

### Geometric parameters (Å, º)

**Table:**

C1—O1	1.3609 (17)	C9—H9A	0.97
C1—C6	1.378 (2)	C9—H9B	0.97
C1—C2	1.421 (2)	C10—C11	1.512 (2)
C2—C3	1.413 (2)	C10—H10A	0.97
C2—C7	1.480 (2)	C10—H10B	0.97
C3—O3	1.3496 (19)	C11—N1	1.4579 (18)
C3—C4	1.389 (2)	C11—H11A	0.97
C4—C5	1.367 (2)	C11—H11B	0.97
C4—H4	0.93	C12—C13	1.359 (2)
C5—C6	1.381 (2)	C12—N1	1.364 (2)
C5—H5	0.93	C12—H12	0.93
C6—H6	0.93	C13—C14	1.396 (2)
C7—O2	1.2406 (18)	C13—H13	0.93
C7—C8	1.490 (2)	C14—C15	1.362 (2)
C8—H8A	0.96	C14—H14	0.93
C8—H8B	0.96	C15—N1	1.357 (2)
C8—H8C	0.96	C15—H15	0.93
C9—O1	1.4297 (17)	O3—H3	0.98 (2)
C9—C10	1.5042 (19)		
			
O1—C1—C6	122.13 (13)	C10—C9—H9B	109.9
O1—C1—C2	116.67 (12)	H9A—C9—H9B	108.3
C6—C1—C2	121.19 (13)	C9—C10—C11	108.84 (12)
C3—C2—C1	116.77 (13)	C9—C10—H10A	109.9
C3—C2—C7	118.82 (13)	C11—C10—H10A	109.9
C1—C2—C7	124.41 (13)	C9—C10—H10B	109.9
O3—C3—C4	116.60 (14)	C11—C10—H10B	109.9
O3—C3—C2	121.99 (15)	H10A—C10—H10B	108.3
C4—C3—C2	121.41 (14)	N1—C11—C10	114.44 (13)
C5—C4—C3	119.40 (14)	N1—C11—H11A	108.7
C5—C4—H4	120.3	C10—C11—H11A	108.7
C3—C4—H4	120.3	N1—C11—H11B	108.7
C4—C5—C6	121.59 (15)	C10—C11—H11B	108.7
C4—C5—H5	119.2	H11A—C11—H11B	107.6
C6—C5—H5	119.2	C13—C12—N1	108.26 (14)
C1—C6—C5	119.62 (15)	C13—C12—H12	125.9
C1—C6—H6	120.2	N1—C12—H12	125.9
C5—C6—H6	120.2	C12—C13—C14	107.29 (15)
O2—C7—C2	119.14 (14)	C12—C13—H13	126.4
O2—C7—C8	117.11 (14)	C14—C13—H13	126.4
C2—C7—C8	123.74 (13)	C15—C14—C13	107.58 (15)
C7—C8—H8A	109.5	C15—C14—H14	126.2
C7—C8—H8B	109.5	C13—C14—H14	126.2
H8A—C8—H8B	109.5	N1—C15—C14	108.19 (15)
C7—C8—H8C	109.5	N1—C15—H15	125.9
H8A—C8—H8C	109.5	C14—C15—H15	125.9
H8B—C8—H8C	109.5	C15—N1—C12	108.68 (13)
O1—C9—C10	108.82 (11)	C15—N1—C11	125.99 (14)
O1—C9—H9A	109.9	C12—N1—C11	125.22 (13)
C10—C9—H9A	109.9	C1—O1—C9	118.35 (11)
O1—C9—H9B	109.9	C3—O3—H3	102.9 (12)
			
O1—C1—C2—C3	179.16 (12)	C3—C2—C7—C8	173.87 (14)
C6—C1—C2—C3	−0.6 (2)	C1—C2—C7—C8	−5.6 (2)
O1—C1—C2—C7	−1.4 (2)	O1—C9—C10—C11	−179.11 (12)
C6—C1—C2—C7	178.83 (13)	C9—C10—C11—N1	170.05 (13)
C1—C2—C3—O3	−179.70 (13)	N1—C12—C13—C14	0.30 (18)
C7—C2—C3—O3	0.8 (2)	C12—C13—C14—C15	−0.2 (2)
C1—C2—C3—C4	0.9 (2)	C13—C14—C15—N1	0.10 (19)
C7—C2—C3—C4	−178.57 (13)	C14—C15—N1—C12	0.08 (18)
O3—C3—C4—C5	179.94 (16)	C14—C15—N1—C11	176.39 (15)
C2—C3—C4—C5	−0.7 (3)	C13—C12—N1—C15	−0.24 (18)
C3—C4—C5—C6	0.1 (3)	C13—C12—N1—C11	−176.59 (14)
O1—C1—C6—C5	−179.71 (14)	C10—C11—N1—C15	104.65 (18)
C2—C1—C6—C5	0.1 (2)	C10—C11—N1—C12	−79.63 (19)
C4—C5—C6—C1	0.2 (3)	C6—C1—O1—C9	0.8 (2)
C3—C2—C7—O2	−5.5 (2)	C2—C1—O1—C9	−179.01 (12)
C1—C2—C7—O2	174.99 (14)	C10—C9—O1—C1	176.69 (12)

### Hydrogen-bond geometry (Å, º)

Cg is is the centroid of the N1/C12–C15 ring.

**Table:**

*D*—H···*A*	*D*—H	H···*A*	*D*···*A*	*D*—H···*A*
O3—H3···O2	0.98 (2)	1.578 (19)	2.498 (2)	153.4 (18)
C5—H5···*Cg*^i^	0.93	2.90	3.641 (2)	138
C11—H11*B*···*Cg*^ii^	0.97	2.74	3.3973 (19)	125

Symmetry codes: (i) *x*, *y*−1, *z*; (ii) −*x*+1, −*y*+2, −*z*.

## References

[bb1] Brandenburg, K. & Berndt, M. (2001). *DIAMOND* Crystal Impact GbR, Bonn, Germany.

[bb2] Burla, M. C., Caliandro, R., Camalli, M., Carrozzini, B., Cascarano, G. L., De Caro, L., Giacovazzo, C., Polidori, G. & Spagna, R. (2005). *J. Appl. Cryst.* **38**, 381–388.

[bb3] Coche-Guerente, L., Cosnier, S., Innocent, C. & Mailly, P. (1995). *Anal. Chim. Acta*, **311**, 23–30.

[bb4] Deronzier, A. & Moutet, J. C. (1996). *Coord. Chem. Rev.* **147**, 339–371.

[bb5] Dong, W. K., Sun, Y. X., Zhao, C. Y., Dong, X. Y. & Xu, L. (2010). *Polyhedron*, **29**, 2087–2097.

[bb6] Farrugia, L. J. (1997). *J. Appl. Cryst.* **30**, 565.

[bb7] Farrugia, L. J. (1999). *J. Appl. Cryst.* **32**, 837–838.

[bb8] Huo, L. H., Cao, L. X., Wang, D. M., Cui, N. N., Zeng, G. F. & Xi, S. Q. (1999). *Thin Solid Films*, **350**, 5–9.

[bb9] Khedkar, S. P. & Radhakrishnan, S. (1997). *Thin Solid Films*, **303**, 167–172.

[bb10] MacDearmid, A. G. (2001). *Rev. Mod. Phys.* **73**, 701–712.

[bb11] Nonius (1998). *COLLECT* Nonius BV, Delft, The Netherlands.

[bb12] Otwinowski, Z. & Minor, W. (1997). *Methods in Enzymology*, Vol. 276, *Macromolecular Crystallography*, Part A, edited by C. W. Carter Jr & R. M. Sweet, pp. 307–326. New York: Academic Press.

[bb13] Ourari, A., Baameur, L., Bouet, G. & Khan, A. M. (2008). *J. Electrochem. Commun.* **10**, 1736–1739.

[bb14] Saraswat, K., Prasad, R. N., Ratnani, R., Drake, J. E., Hursthouse, M. B. & Light, M. E. (2006). *Inorg. Chim. Acta*, **359**, 1291–1295.

[bb15] Sheldrick, G. M. (2008). *Acta Cryst.* A**64**, 112–122. 10.1107/S0108767307043930 18156677

[bb16] Smith, G. A., Tasker, P. A. & White, D. J. (2003). *Coord. Chem. Rev.* **241**, 61–85.

[bb17] Srinivasan, K., Michaud, P. & Kochi, J. K. (1986). *J. Am. Chem. Soc.* **108**, 2309–2320. 10.1021/ja00269a029 22175576

[bb18] Wu, S. & Lu, S. (2003). *J. Mol. Catal. A*, **198**, 29–38.

